# A Simple Score to Identify Super-Responders to Sacubitril/Valsartan in Ambulatory Patients With Heart Failure

**DOI:** 10.3389/fphys.2021.642117

**Published:** 2021-02-18

**Authors:** Carles Moliner-Abós, Diana Mojón Álvarez, Mercedes Rivas-Lasarte, Laia Carla Belarte, Julia Pamies Besora, Eduard Solé-González, Paula Fluvià-Brugues, Isabel Zegrí-Reiriz, Laura López López, Vicens Brossa, Maria José Pirla, Nuria Mesado, Sonia Mirabet, Eulàlia Roig, Jesús Álvarez-García

**Affiliations:** ^1^Cardiology Department, Hospital de la Santa Creu i Sant Pau, IIB-SantPau, CIBERCV, Universidad Autónoma de Barcelona, Barcelona, Spain; ^2^Cardiology Department, Hospital del Mar, Barcelona, Spain; ^3^The Zena and Michael A. Wiener Cardiovascular Institute, Icahn School of Medicine at Mount Sinai, New York, NY, United States

**Keywords:** sacubitril/valsartan, heart failure, super-response, score, cardiac remodeling

## Abstract

**Introduction:**

Sacubitril/valsartan (SV) promotes cardiac remodeling and improves prognosis in patients with heart failure (HF). However, the response to the drug may vary between patients and its implementation in daily clinical practice has been slower than expected. Our objective was to develop a score predicting the super-response to SV in HF outpatients.

**Methods:**

This is a retrospective analysis of 185 consecutive patients prescribed SV from two tertiary hospitals between September 2016 and February 2018. Super-responder was defined as a patient taking the drug and (i) without HF admissions, death, or heart transplant, and (ii) with a ≥50% reduction in NT-proBNP levels and/or an increase of ≥10 points in LVEF in a 12-month follow-up period after starting SV. Clinical, echocardiographic, ECG, and biochemical variables were used in a logistic regression analysis to construct a score for super-response to SV which was internally validated using bootstrap method.

**Results:**

Out of 185 patients, 65 (35%) fulfilled the super-responder criteria. Predictors for super-response to SV were absence of both previous aldosterone antagonist and diuretic treatment, NYHA I-II class, female gender, previous 1-year HF admission, and sinus rhythm. An integrating score distinguished a low- (<25%), intermediate- (∼46%), and high-probability (>80%) for 1-year super-response to SV. The AUC for the model was 0.72 (95%CI: 0.64–0.80), remaining consistent after internal validation.

**Conclusion:**

One-third of our patients presented a super-response to SV. We propose an easy-to-calculate score to predict super-response to SV after 1-year initiation based on variables that are currently assessed in clinical practice.

## Introduction

Sacubitril/valsartan (SV) is the first agent of the angiotensin receptor–neprilysin inhibitor (ARNI) drug class (Singh et al., 2017). In the PARADIGM−HF trial, ARNI compared to enalapril reduced the risk of cardiovascular death or heart failure (HF) hospitalization by 20%. Moreover, the risks of all−cause, cardiovascular, and sudden cardiac death were also significantly reduced by SV (McMurray et al., 2014). Therefore, both American and European guidelines on HF recommend SV for symptomatic HF patients with reduced ejection fraction (HFrEF) (Ponikowski et al., 2016; Yancy et al., 2017).

Although the benefit of SV was consistent across the clinical spectrum of HF (McMurray et al., 2014), the response to the drug may vary between patients from real life and those from clinical trial. In fact, the study population of the trial represented a subset of a larger cohort eligible for the initial run-in period ensuring tolerability of target doses of both enalapril and ARNI before randomization (Desai et al., 2016). Previous studies (Pellicori et al., 2017; Martens et al., 2018), and more recently, the Prospective Study of Biomarkers, Symptom Improvement, and Ventricular Remodeling During SV Therapy for Heart Failure (PROVE-HF) study (Januzzi et al., 2019) have showed that SV promotes reverse myocardial remodeling. Moreover, a sub-analysis of this study showed that patients with a higher decrease in NT-proBNP levels or in left ventricular end diastolic diameter during the first 6 months after SV initiation had a lower rate of HF hospitalizations or death in the following months (Januzzi et al., 2020). However, the identification of such predictors of super-response to ARNI is insufficient so far (Martens et al., 2018; Nakou et al., 2018; Moliner-Abós et al., 2019; Vicent et al., 2019; Chang et al., 2020; Díez-Villanueva et al., 2020). In addition, since its approval in late 2015 the implementation of SV in daily clinical practice has been poor, partially due to high cost (Di Tano and Bettari, 2017; DeVore et al., 2018; Sangaralingham et al., 2018; Kahn et al., 2020). So, the development of tools aimed to recognize patients with a very favorable response to specific drugs could improve the efficiency in a setting where pharmacological treatment is increasingly complex and expensive.

Hence, we conducted a study aimed to determine the prevalence and clinical predictors of super-response to SV.

## Materials and Methods

### Study Population

This study includes a cohort of all consecutive patients attended at the HF clinics from two tertiary referral centers in Barcelona (Spain) since September 2016 to February 2018 in which SV was introduced. The clinical criteria for initiating drug were (i) symptomatic HF defined as New York Heart Association (NYHA) class II–IV, (ii) left ventricular ejection fraction (LVEF) ≤ 40% measured by echocardiography, and (iii) pretreatment according to the current European Society of Cardiology (ESC) guidelines (Ponikowski et al., 2016) (including ACEI or ARB). All procedures performed in the study were in accordance with the ethical standards of the institutional research committee and with the Helsinki declaration. Due to the retrospective nature of the study, it was considered that informed consent was not required.

### Study Variables

Data were collected using the electronic health record of both hospitals. The following clinical variables were gathered at study inclusion and during the follow-up period: demographic and previous clinical history, NYHA functional class, systolic blood pressure, laboratory blood tests including NT-proBNP within the previous 30 days before SV initiation, ECG, echocardiography within the previous 6 months, and pharmacological and non-pharmacological treatment.

### Super-Response to SV and Follow-Up

After initiation of SV, the frequency of the follow-up visits was performed at the discretion of the attending cardiologist. Most of the patients were visited every 2–4 weeks at the HF clinic of each center, with renal function, potassium, and clinical status check. Once the drug was up-titrated until maximum tolerated dose, a new determination of NT-proBNP and echocardiography were usually done. The incident HF follow-up duration was calculated as the time from starting SV until the time of censoring: death, heart transplantation, or a 12-month complete period. Over a similar follow-up duration, the number of HF admissions before the initiation of SV was calculated. Super-responder to SV was defined as a patient taking the drug and (i) without HF admissions, death, or heart transplant and (ii) with a ≥50% reduction in NT-proBNP levels (Zile et al., 2016) and/or an increase of ≥10 points in LVEF (Januzzi et al., 2019) in a 12-month follow-up period after starting the SV. The primary objective was to determine the prevalence and predictors of super-response to SV.

### Statistical Analysis

Continuous variables were expressed as the mean ± standard deviation (SD) or as median (interquartile range) and were compared with the Student’s *t*-test, log-rank test. Categorical variables were expressed as percentages and compared with the χ^2^ or Fisher’s exact tests.

Variable selection was performed using logistic regression models, with super-response to SV as a dichotomous dependent variable. In order to find the best predictive model with the highest area under the receiving operating characteristic (ROC) curve (AUC), exploratory analyses were performed including clinical meaningful variables showing a significant level in the univariate analysis (*p*-value < 0.2) and prioritizing parsimony. The internal validity of the final predictive model was tested for 15 bootstrap re-samples, using the “CVAUROC” package for Stata (Luque-Fernandez et al., 2019). The calibration of the model was assessed by the Hosmer–Lemeshow test for goodness of fit and plotting the observed frequencies against the expected probabilities. Finally, a score of super-response was proposed as the addition of the β coefficients of each predictor multiplied by 10 and rounded to the nearest integer number. Mean crossvalidated sensitivity and specificity for each category of the score were presented. A two-sided *p*-value < 0.05 was considered statistically significant. All the analyses were performed using STATA software (v. 13.1).

## Results

### Prevalence of Super-Responders to Sacubitril/Valsartan

Out of 185 patients, 65 (35.1%) fulfilled the super-responder criteria. Of them, 29 patients presented an NT-proBNP reduction of 50% or more, 26 improved LVEF at least 10 points, and, finally, 10 patients accomplished both conditions. Overall, super-responders were more frequent in sinus rhythm and without aldosterone antagonists or diuretics at the time of SV initiation than standard responders. Although non-significant statistically, clinical characteristics such as women, NYHA I-II class, less dilated left ventricle, and without implantable cardioverter defibrillator were more frequent among super-responders. Lastly, previous HF admission rate during the past year was higher in the super-responder group. After a 1-year follow-up, super-responders presented a better NYHA class, higher LVEF, lower plasma levels of NT-proBNP, and similar renal function with higher doses of SV compared to those with a standard response. Table 1 summarizes the clinical characteristics of the study population.

**TABLE 1:** Clinical characteristics of the study population.

	**Super-responders (*N* = 65)**	**Standard responders (*N* = 120)**	***P*-value**	**Study population (*N* = 185)**
Age, years	65 (13)	67 (12)	0.354	67 (12)
Sex, female	20 (31)	26 (22)	0.171	46 (25)
Ischemic etiology	30 (46)	59 (49)	0.695	89 (48)
NYHA class			0.090	
I	2 (3)	2 (2)		4 (2)
II	47 (72)	67 (56)		114 (61)
III	16 (25)	49 (40)		65 (35)
IV	0 (0)	2 (2)		2 (1)
LVEF, %	31 (6)	31 (7)	0.708	31 (6)
LVEDD, mm	60 (9)	63 (9)	0.077	62 (9)
ECG rhythm			0.027	
Sinusal	47 (72)	66 (55)		113 (61)
Atrial fibrillation	18 (28)	46 (39)		64 (35)
Pacemaker	0 (0)	7 (6)		7 (4)
SBP, mmHg	124 (15)	121 (20)	0.242	122 (18)
NT-proBNP, ng/L	1694 (888–4103)	1817 (898–3917)	0.982	1844 (891–4104)
Creatinine, mg/dL	1.1 (0.4)	1.1 (0.3)	0.651	1.1 (0.3)
GFR, mL/min/1.73 m^2^	70 (20)	68 (17)	0.637	69 (18)
GFR < 60 mL/min/1.73 m^2^	22 (35)	48 (40)	0.475	70 (38)
Potassium, mEq/L	4.4 (0.5)	4.5 (0.5)	0.340	4.5 (0.5)
ICD	16 (25)	45 (38)	0.075	61 (33)
CRT	8 (12)	20 (17)	0.417	28 (15)
Mitraclip	2 (3)	4 (3)	0.925	6 (3)
Levosimendan	10 (15)	15 (13)	0.584	25 (14)
ACEi/ARB	58 (89)	110 (92)	0.584	168 (91)
Beta-blockers	63 (97)	111 (93)	0.225	174 (94)
MRA	39 (60)	96 (80)	0.003	135 (73)
Ivabradine	10 (16)	21 (18)	0.728	31 (17)
Diuretics	44 (68)	99 (83)	0.022	143 (73)
Initial dose ARNI, mg			0.517	
24/26	41 (63)	85 (71)		126 (68)
49/51	22 (34)	33 (28)		55 (30)
97/103	2 (3)	2 (2)		4 (2)
HF admission in last year	38 (59)	53 (44)	0.063	91 (49)
**After 1 year follow-up**				
NYHA class			<0.001	
I	22 (34)	16 (16)		38(23)
II	39 (60)	54 (53)		93 (56)
III	4 (6)	29 (28)		33 (20)
IV	0 (0)	3 (3)		3 (1)
LVEF,%	42 (12)	33 (9)	<0.001	36 (11)
≥10 points LVEF	36 (56)	10 (10)	<0.001	46 (29)
NT-proBNP, ng/L	696 (259–1802)	1766 (850–3120)	<0.001	1324 (574–2513)
≥50% of change in NT-proBNP	39 (66)	18 (16)	<0.001	57 (33)
Creatinine, mg/dL	1.2 (0.4)	1.3 (0.7)	0.104	1.3 (0.6)
GFR, mL/min/1.73 m^2^	65 (21)	61 (20)	0.221	63 (20)
Final dose ARNI, mg			<0.001	
24/26	11 (17)	24 (20)		35(19)
49/51	14 (22)	18 (15)		32 (17)
97/103	40 (62)	41 (34)		81 (44)
Discontinuing	0 (0)	37 (31)		37 (20)
1-year HF admission	0 (0)	50 (42)	<0.001	50 (27)
1-year heart transplant	0 (0)	8 (7)	<0.001	8 (4)
1-year mortality	0 (0)	24 (20)	<0.001	24 (13)

*Results are expressed in number (%), mean (SD), or median (IQR). List of abbreviations: NYHA, New York Heart Association; LVEF, left ventricular ejection fraction; LVEDD, left ventricular end diastolic diameter; SBP, systolic blood pressure; NT-proBNP, NT amino terminal propeptide brain natriuretic peptide; GFR, glomerular filtration rate; ICD, implantable cardioverter defibrillator; CRT, cardiac resynchronize therapy; ACEi, angiotensin cardioverter enzyme inhibitor; ARB, aldosterone receptor antagonist; MRA, mineraloid receptor antagonist; ARNI, aldosterone receptor neprilysin inhibitor; HF, heart failure.*

### Predictors for Super-Response: The AWARD-Class Score

**FIGURE 1:**
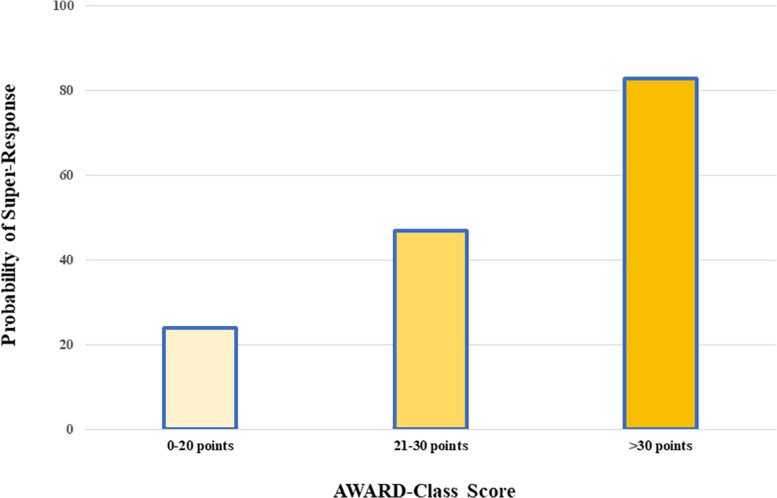
Probability of super-response to sacubitril/valsartan according to the AWARD-Class score categories.

After a multivariate logistic analysis, the absence of previous aldosterone antagonist treatment (OR 2.3, 95% CI: 1.1–4.8) and NYHA I-II class (OR 2.3, 95% CI: 1.1–4.8) were the only independent predictors of super-response to SV. Along with these two factors, the model which predicted best the super-response to SV included gender, presence of HF admission over the past year, ECG rhythm, and presence of diuretics (AUC 0.720; 95% CI: 0.644–0.797). In order to build a score able to predict the probability of super-response for a given patient, we assigned a scale of 41 points based on the β-coefficient of each variable (Table 2). This score—the AWARD-Class score—allowed the estimation of the chance of super-response to SV, as illustrated in Figure 1. Indeed, this score distinguished a low- (24% event rate), intermediate- (47% event rate), and high-probability groups (83% event rate) for 1-year follow-up after SV initiation. Table 3 resumes the prognostic performance of the AWARD-Class score in each category.

**TABLE 2:** The AWARD-Class score.

	**β coefficient**	**Points**	**OR (95% confidence interval)**	***P*-value**
**A**ntialdosteronic naive	0.836	8	2.3 (1.1–4.8)	0.024
**W**oman	0.536	5	1.7 (0.8–3.6)	0.161
HF **A**dmission in last year	0.601	6	1.8 (0.9–3.6)	0.088
Sinus **R**hythm	0.692	7	2.0 (1.0–4.0)	0.052
**D**iuretics naive	0.743	7	2.1 (1.0–4.5)	0.059
I-II NYHA **Class**	0.835	8	2.3 (1.1–4.8)	0.025

*List of abbreviations: OR, odds ratio; HF, heart failure; NYHA, New York Heart Association.*

**TABLE 3:** Prognostic performance of the AWARD-Class score categories.

	**Sensitivity**	**Specificity**	**PPV**	**NPV**
Low score (0–20 points)	88%	32%	29%	89%
Intermediate score (21–30 points)	37%	90%	77%	62%
High score (≥31 points)	0%	100%	100%	17%

*List of abbreviations: PPV, positive predictive value; NPV, negative predictive value.*

### Internal Validation and Calibration of the Model

**FIGURE 2:**
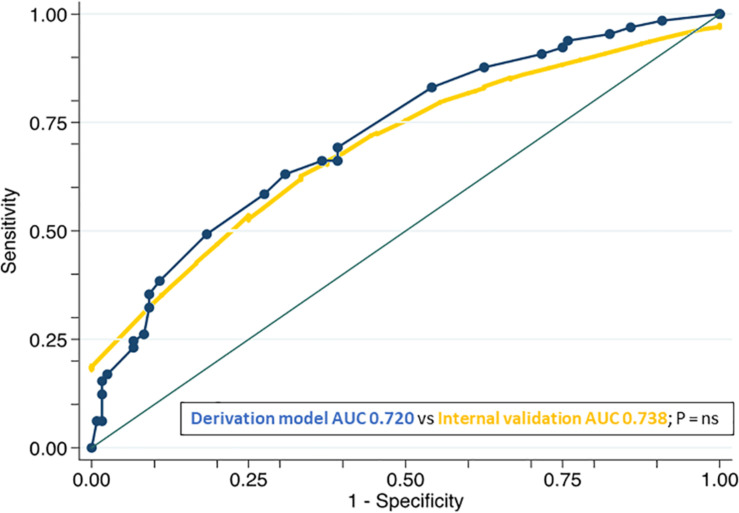
Comparison of the ROC curves of the derivation (curve containing dots) and internal validation models. List of abbreviations: ROC, receiver operating characteristic; AUC, area under the curve; P, *p*-value; NS, non-significance.

**FIGURE 3:**
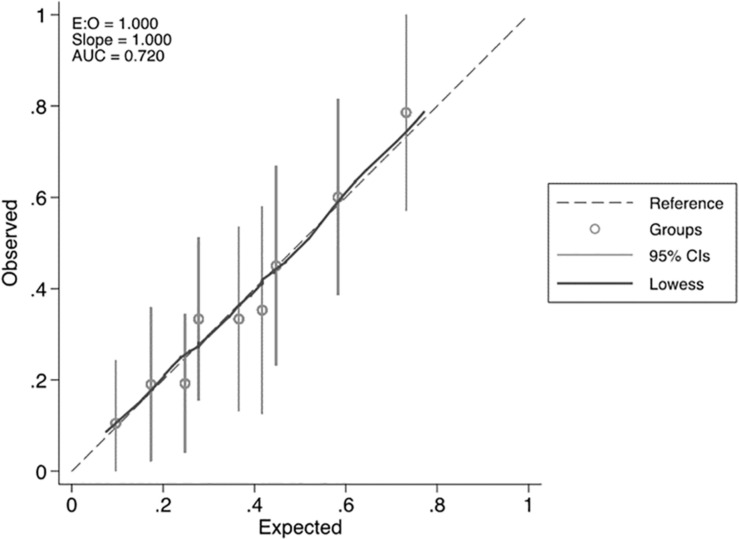
Calibration plot of the AWARD-Class score for super-response to sacubitril/valsartan.

After the bootstrap sampling, the AUC for this model was 0.738 (95% CI 0.616–0.792), which was non-statistically different compared to the AUC of the derivation cohort (Figure 2). Calibration of the score was fairly good as shown by the non-significant Hosmer–Lemeshow test (*P* = 0.820) and the calibration plot (Figure 3).

## Discussion

### Main Findings

Up to 35% of patients from two different tertiary hospitals fulfilled the super-responder criteria to SV. This study proposes a simple score that predicts super-response to SV after 1-year initiation. Moreover, the score allows discrimination between low-, intermediate-, and high-probability of super-response based on variables that are currently assessed in clinical practice.

### Predictors of Super-Response to Sacubitril/Valsartan

Only a few studies have indicated possible predictors for a favorable response to SV in ambulatory patients with HF (Martens et al., 2018; Nakou et al., 2018; Vicent et al., 2019; Chang et al., 2020; Díez-Villanueva et al., 2020) (Table 4). These are observational cohort studies, most reflecting single-center experiences, and with a follow-up of less than 1 year after the start of the drug. The favorable response to SV was defined as the evidence of a positive cardiac remodeling assessed by echocardiography or a significant improvement in the functional capacity of patients. Two studies agreed that higher doses of SV were more likely to observe a favorable response (Martens et al., 2018; Chang et al., 2020). Other predictors proposed were female sex (Vicent et al., 2019), the absence of an ICD (Díez-Villanueva et al., 2020), the non-ischemic etiology of HF (Chang et al., 2020), the smaller diameter of the LV (Chang et al., 2020), and the plasmatic levels of troponin (Nakou et al., 2018). In this regard, our study coincides in that women were significantly more frequent “super-responders” than men. This fact has been equally observed in both the PARAGON-HF trial (McMurray et al., 2020) and in a pooled analysis with the results of the PARADIGM trial (Solomon et al., 2020), where SV seemed to reduce the risk of HF hospitalization more in women than in men in higher values of LVEF, and also with other therapeutic options for patients with low LVEF such as CRT (Costanzo, 2017). Smaller LV end diastolic diameter and the absence of previous ICD were also more prevalent in our super-responder population; however, these factors were not part of the best equation to predict the super-response to SV. Instead, the AWARD-Class score proposes that aldosterone antagonist-naive patients and those with a previous HF-admission during last year—probably reflecting subjects with a shorter history of HF—along with better NYHA class and the absence of diuretics—translating a less advanced HF stage—are the best predictors for a super-response to SV in our study population. Supporting the concept of early initiation of SV, recent randomized clinical trials have shown that SV initiation during or early after an HF hospitalization—even in *de novo* HF—conferred a better prognosis compared to previous standard treatment (Velazquez et al., 2019; Wachter et al., 2019b; Senni et al., 2020). Moreover, our model has been developed integrating populations from two different hospitals and establishing as a mandatory criterion for super-response the absence of hard clinical events such as HF readmission, heart transplantation, or death over the next year, in addition to positive cardiac remodeling and/or a significant reduction of NT-proBNP levels. Importantly, this simple score is based on variables routinely collected in the daily clinical practice and allows stratifying into three groups according to the probability of super-response. Indeed, if a patient scores more than 30 points, the probability of not suffering any clinical event and recovering at least 10 points of LVEF and/or halve at least their previous levels of natriuretic peptides after 1 year would be *sky high*, given that this group shows a specificity and a positive predictive value of 100% after internal validation (Table 3).

**TABLE 4:** Predictors for a favorable response to SV in previous studies.

**References**	**N**	**Design**	**FU**	**Response**	**Predictors**
Martens et al., 2018	141	Single-center	∼3 m	Cardiac remodeling by echo	High dose of SV
Nakou et al., 2018	48	Single-center	6 m	Functional capacity by CPET	Troponin-I
Díez-Villanueva et al., 2020	249	Multi-center	∼7 m	Cardiac remodeling by echo	Non-ICD
Vicent et al., 2019	427	Multi-center	∼7 m	Functional capacity by NYHA	Females
Chang et al., 2020	437	Single-center	12 m	Cardiac remodeling by echo	Non-ischemic, ↓LVEDD, high dose of SV

*List of abbreviations: SV, sacubitril/valsartan; FU, follow-up; m, months; CPET, cardio-pulmonary exercise testing; ICD, implantable cardioverter defibrillator; LVEDD, left ventricular end diastolic diameter.*

### Clinical Implications

In the outpatient environment, this score should provide the opportunity to identify those patients who would most benefit from SV. We consider that its use could imply several clinical benefits, especially in three scenarios. First, it would help its implementation in daily clinical practice, which until now has been slower than expected in different countries (DeVore et al., 2018; Wachter et al., 2019a; de Frutos et al., 2020). Selecting patients with a super-response could be particularly helpful in health systems where resources are limited (Krittayaphong and Permsuwan, 2018; Kahn et al., 2020). Second, the identification of a subgroup of patients with an excellent 1-year prognosis (without events and positive cardiac remodeling) would allow in turn to differ the potential indication of expensive and invasive complex therapies, such as CRT or ICD (Zacà, 2018), especially in patients with non-ischemic cardiomyopathy (Jilek et al., 2020). And third, the AWARD-Class score fuels the debate on the timing that SV should be started within the treatment algorithm of patients with HF and reduced LVEF (Ambrosy et al., 2019; Escobar et al., 2019).

### Study Limitations

Several limitations of this study have to be stated. This was a retrospective observational study with a relatively small number of patients. Although the AWARD-Class score includes a wide range of relevant variables of HF, we did not collect specific information about other comorbidities or psychosocial factors. Both hospitals comprised patients from the same geographic area and mostly Caucasians; thus our model would need further validation in different populations. In addition, we recognize that the “super-responder” concept has been defined arbitrarily, but we have chosen the conditions based on the results of the two largest studies evaluating NT-proBNP change (Zile et al., 2016) and cardiac remodeling (Januzzi et al., 2019). Finally, further studies are warranted to test the performance of this score at longer follow-ups and in different clinical scenarios such as acute HF hospitalization or with the addition of new drugs to the HFrEF armamentarium (McMurray et al., 2019; Ryan and Maron, 2020; Santos-Gallego et al., 2020; Teerlink et al., 2020).

## Conclusion

The prevalence of super-responders to SV was of 35% in our study population. A simple score based on precise variables that are currently assessed in clinical practice to predict super-response to SV after 1-year initiation is proposed and should be validated in different populations.

## Data Availability Statement

The raw data supporting the conclusions of this article will be made available by the authors, without undue reservation.

## Ethics Statement

The studies involving human participants were reviewed and approved by Hospital del Mar, Barcelona. Written informed consent for participation was not required for this study in accordance with the national legislation and the institutional requirements.

## Author Contributions

MR-L, JÁ-G, LB, ES-G, SM, and ER conceptualized or designed the work. CM-A, DM, JP, PF-B, IZ-R, LL, VB, MP, and NM contributed to data acquisition. CM-A, MR-L, JÁ-G, LB, and ES-G analyzed or interpreted the data. CM-A, MR-L, and JÁ-G drafted the work. All the authors substantially contributed in the critical revision of the manuscript, ensured the accuracy, and approved the final version.

## Conflict of Interest

JÁ-G, SM, and ER have received speaker honorariums from Novartis. JÁ-G and SM have received speaker honorariums from Rovi. MR-L and PF-B have received an educational grant from Novartis. The remaining authors declare that the research was conducted in the absence of any commercial or financial relationships that could be construed as a potential conflict of interest.
